# Targeting NETosis: nature’s alarm system in cancer progression

**DOI:** 10.20517/cdr.2024.24

**Published:** 2024-07-19

**Authors:** Yixia Liang, Guo Wu, Jiabao Tan, Xiaoyun Xiao, Linbin Yang, Phei Er Saw

**Affiliations:** ^1^Guangdong Provincial Key Laboratory of Malignant Tumor Epigenetics and Gene Regulation, Guangdong-Hong Kong Joint Laboratory for RNA Medicine, Medical Research Center, Sun Yat-Sen Memorial Hospital, Sun Yat-Sen University, Guangzhou 510120, Guangdong, China.; ^2^Nanhai Translational Innovation Center of Precision Immunology, Sun Yat-Sen Memorial Hospital, Foshan 528200, Guangdong, China.; ^3^Department of Ultrasound, Sun Yat-sen Memorial Hospital, Sun Yat-sen University, Guangzhou 510120, Guangdong, China.; ^4^Breast Tumor Center, Guangdong Provincial Key Laboratory of Malignant Tumor Epigenetics and Gene Regulation, Medical Research Center, Sun Yat-sen Memorial Hospital, Sun Yat-sen University, Guangzhou 510120, Guangdong, China.; ^#^Authors contributed equally.

**Keywords:** Neutrophils, neutrophils extracellular traps (NETs), NETosis, cancer progression, cancer therapeutics

## Abstract

Neutrophils are recognized active participants in inflammatory responses and are intricately linked to cancer progression. In response to inflammatory stimuli, neutrophils become activated, releasing neutrophils extracellular traps (NETs) for the capture and eradication of pathogens, a phenomenon termed NETosis. With a deeper understanding of NETs, there is growing evidence supporting their role in cancer progression and their involvement in conferring resistance to various cancer therapies, especially concerning tumor reactions to chemotherapy, radiation therapy (RT), and immunotherapy. This review summarizes the roles of NETs in the tumor microenvironment (TME) and their mechanisms of neutrophil involvement in the host defense. Additionally, it elucidates the mechanisms through which NETs promote tumor progression and their role in cancer treatment resistance, highlighting their potential as promising therapeutic targets in cancer treatment and their clinical applicability.

## INTRODUCTION

Evidence substantiates a strong link between inflammation and cancer development. Inflammatory cells and cytokines actively contribute to the inflammatory tumor microenvironment (TME), which exhibits a significant degree of heterogeneity, thereby exerting a substantial influence on the malignancy of tumors and the response to therapeutic interventions^[1-3]^. Various innate immune cells within the TME include tumor-associated macrophages (TAMs), tumor-associated neutrophils (TANs), and myeloid-derived suppressor cells (MDSCs)^[4-7]^. While the role of TAMs in tumor biology has become relatively well understood over the past two decades, the contribution of TANs to these intricate processes remains largely elusive. It is necessary to elucidate the role of TANs in inflammation and cancer progression.

Neutrophils constitute 50%-70% of circulating white blood cells^[8]^. Being the predominant immune cells within the innate immune system, neutrophils assume a pivotal role in defending against bacterial pathogenic infections by generating neutrophils extracellular traps (NETs) through a process known as NETosis. NETs are considered to be based on DNA, which is embedded with histone, neutrophil elastase (NE), myeloperoxidase (MPO), calprotectin, matrix metalloproteinases (MMP), cathepsin G (CG), and other granular proteins, proteolytic enzymes, antibacterial peptides, and histones^[9]^. The process of NET production by neutrophils is called NETosis. However, beyond their established role in host defense, accumulating evidence underscores the pivotal contribution of NETs to both cancer progression and resistance to treatment. Chemotherapy, immunotherapy, and radiation therapy (RT) stand as cornerstone modalities in cancer treatment; thus, the development of novel strategies to mitigate resistance to these therapies is of paramount importance. In this review, we summarize the mechanisms underlying neutrophils in host defense and their intricate interactions within the TME. We provide insights into the mechanisms through which NETs foster tumor progression and contribute to resistance against cancer therapies. These findings underscore the potential of NETs as promising therapeutic targets in cancer and emphasize their clinical applicability.

## TUMOR-ASSOCIATED NEUTROPHILS

Neutrophils constitute 50%-70% of white blood cells in the bloodstream^[8]^, with a circulating half-life of approximately 7-10 h in both humans and mice^[10]^. Research has indicated that tumor cells secrete cytokines, such as granulocyte colony-stimulating factor (G-CSF), interleukin-1β (IL-1β), interleukin-6 (IL-6), and tumor necrosis factor (TNF), which are believed to extend the lifespan of neutrophils^[11,12]^. Neutrophils, as integral members of the innate immune system, play a pivotal role in the host’s defense against infections and tissue damage. However, the sustained infiltration of neutrophils serves as a hallmark of chronic inflammation. Within the context of chronic inflammation, immunosuppressive cells are recruited, facilitating the formation of the TME, ultimately promoting the initiation and progression of tumors. Consequently, tumors are often characterized as “wounds that do not heal”. For instance, inflammatory bowel disease may lead to the onset of colon cancer^[13,14]^, while non-alcoholic fatty liver disease or chronic hepatitis heightens the risk of hepatocellular carcinoma (HCC)^[15]^.

Within the physiological state of the human body, more than 1 × 10^11^ neutrophils are generated daily^[16]^. These neutrophils originate in the bone marrow, enter the bloodstream, migrate to sites of inflammation or infection to function, and are eventually cleared by macrophages in the body^[17]^. Concurrently, research has indicated an elevated neutrophil count in the blood of cancer patients compared to healthy individuals^[18,19]^. Thus, the neutrophil-to-lymphocyte ratio (NLR) emerges as a potential biomarker across various cancer types^[20]^. However, due to differences in cancer types, staging, treatment methods, and survival outcomes, the thresholds used to define “high” NLR vary^[20]^. For instance, in patients with malignant mesothelioma, an NLR > 5 is associated with poorer prognosis^[21]^. Similarly, NLR is an independent predictor of short- and long-term mortality in breast cancer patients with an NLR > 3.3^[22]^. A systematic review and meta-analysis of 22 solid tumors found that the median cut-off for NLR was 4^[18]^.

The concept of TANs has garnered increasing attention, indicating a dual role in both promoting and suppressing tumors within the TME. In 2009, Fridlender *et al.* introduced TAN polarization, labeling TANs with antitumor properties as N1 and those with pro-tumor properties as N2^[23]^. They demonstrated that tumors drive the phenotypic shift of TANs toward N2 by expressing transforming growth factor beta (TGF-β). In mouse tumor models, treatment with a TGF-β inhibitor increased the recruitment and activation of antitumor phenotype TANs^[23]^. Beyond tumor cells, other cells in the TME also influence neutrophil polarization. In gastric cancer, gastric cancer-derived mesenchymal stem cells (GC-MSCs) produce IL-6, which, through the IL-6-STAT3-extracellular signal-regulated kinase (ERK)1/2 pathway, steers neutrophils toward the N2 phenotype^[24]^.

While the concept of TAN polarization is widely acknowledged in the research community, it has primarily been explored in mouse models. At present, some distinct markers associated with N1 and N2 phenotypes observed in mice lack clear relevance to human oncology. Neutrophils exhibit remarkable plasticity in the TME, and the emergence of single-cell transcriptomics has revealed a spectrum of neutrophil states in cancer^[25]^. Researchers have delineated five neutrophil subgroups in lung cancer patients (human N1-5) and six subgroups in tumor-bearing mice (mouse N1-6)^[25]^. Furthermore, it was observed that the proportion of neutrophil subgroups differs between tumor-free and tumor-bearing mice. Therefore, the initial binary categorization of neutrophils in tumors through the N1-N2 TANs classification may oversimplify the complexity of neutrophil biology. With the further advancement of transcriptomics in the future, the phenotype and function of neutrophils in the TME may receive additional clarification.

## DEFENSE MECHANISMS OF NEUTROPHILS

**Figure 1:**
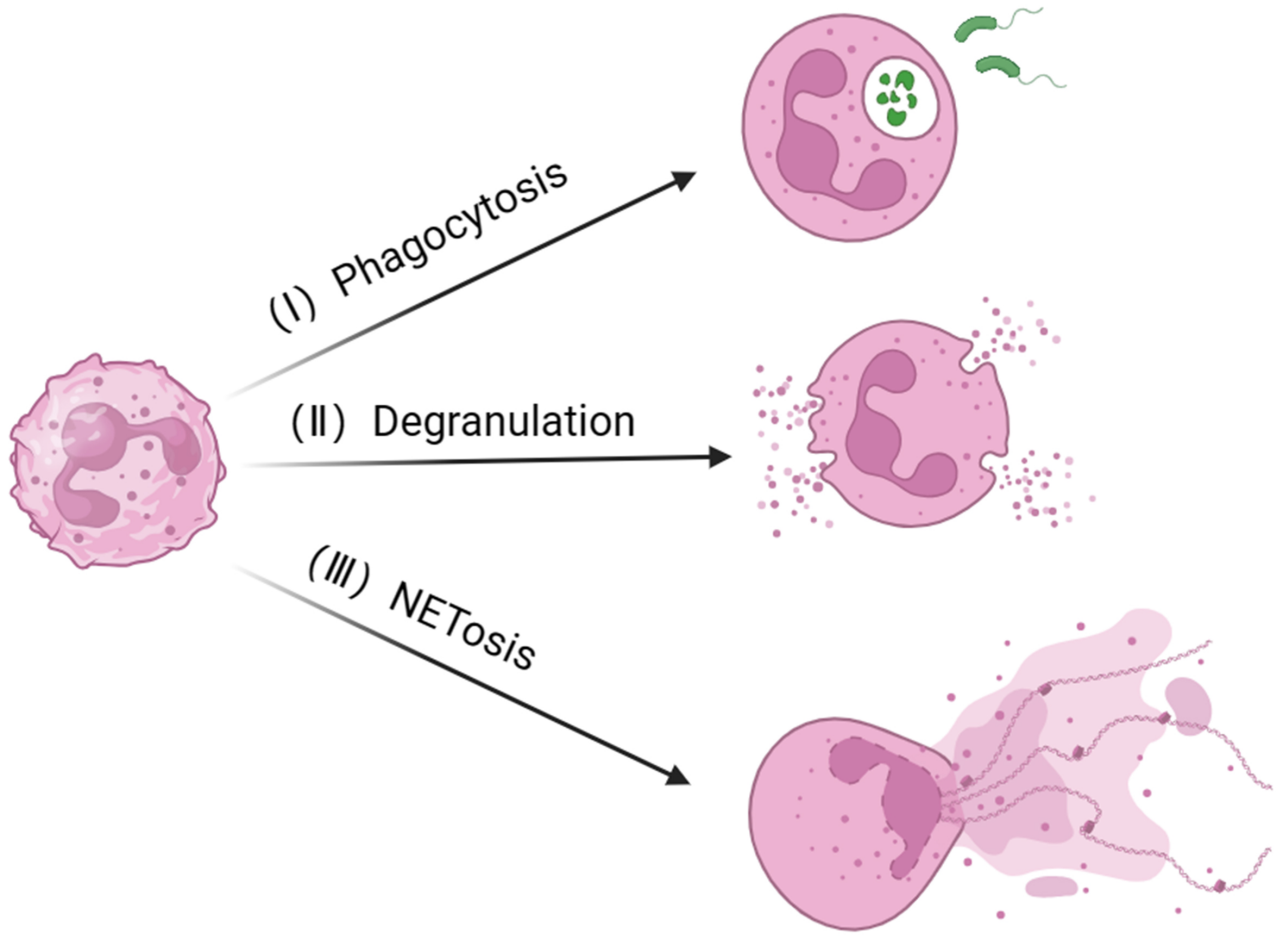
Three defense functions of neutrophils: phagocytosis, degranulation, and NETosis. (I) Phagocytosis: Neutrophils engulf invading pathogens, releasing cytoplasmic granules (such as antimicrobial peptides) upon contact with the engulfed pathogen, thereby facilitating the degradation and elimination of the pathogen; (II) Degranulation: Activated neutrophils release granules stored in the cytoplasm into the extracellular space, acting on extracellular pathogens, leading to their destruction and clearance; (III) NETosis: Activated neutrophils release NETs composed of proteins (such as histones, NE, and MPO) and DNA, which physically capture and eliminate pathogens. Created with BioRender.com (accessed on 30 June 2022). NETs: Neutrophils extracellular traps; NE: neutrophil elastase; MPO: myeloperoxidase; DNA: deoxyribonucleic acid.

Neutrophils, as crucial components of the immune system, play a pivotal role in host defense^[26]^. The defense functions of neutrophils primarily encompass three mechanisms: phagocytosis, degranulation, and NETosis [Figure 1].

### Phagocytosis

Phagosomes physically engulf pathogens and subsequently merge with granules within neutrophils, thereby acquiring antimicrobial enzymes and the NADPH oxidase complex (NOX)^[27]^. NOX generates superoxide molecules, which act on MPO to produce biotoxic substances, such as hypochlorous acid^[28,29]^. Notably, neutrophils exhibit a phagocytic killing capacity against pathogens that is one to two orders of magnitude greater than other immune cells, including macrophages^[30]^.

### Degranulation

Neutrophils can also employ degranulation to combat pathogens, a process where granules within neutrophils fuse with the plasma membrane, releasing soluble proteins into the extracellular space^[31]^. Mature neutrophils possess three types of granules, each releasing distinct proteins in a sequential manner^[32]^. First, tertiary granules, such as Arginase I and MMP-9, are released. Subsequently, secondary granules are discharged, containing proteins like lactoferrin, matrix metalloprotein-8, β2-microglobulin, haptoglobin, and more. Finally, primary granules, also known as azurophilic granules, release a variety of pro-inflammatory and antimicrobial proteins, including MPO, NE, defensins, serine proteases, proteinase 3, CG and C, and bactericidal permeability-increasing protein (BPI). It is noteworthy that the proteins stored in secondary and tertiary granules are inactive and in the form of zymogens, whereas the proteins in primary granules have undergone proteolytic processing to become active before storage^[33]^.

### NETosis

Although phagocytosis and degranulation are the primary defense mechanisms of neutrophils, when pathogens are too large to be engulfed, neutrophils are activated and release NETs to physically capture and kill them. The phenomenon was first observed in 1996, when researchers discovered that neutrophils rapidly die upon phorbol 12-myristate 13-acetate (PMA) treatment through a process distinct from the typical morphological changes and DNA degradation seen in apoptosis or necrosis^[34]^. This process, termed NETosis, was officially named and reported in 2004^[35]^. The research found that stimulating neutrophils with interleukin-8 (IL-8), PMA, or lipopolysaccharide (LPS) leads to neutrophil activation and NET release. NETs were described as three-dimensional mesh structures composed of granule proteins, proteinases, antimicrobial peptides, and DNA^[35]^. Furthermore, studies have revealed that NETs not only provide a localized high concentration of antimicrobial substances but also serve as a physical barrier to impede pathogen dissemination. As a defense mechanism of the host, NETs are effective against viral and fungal infections^[36]^. However, with the advancement of research, it has become evident that NETs can act as a double-edged sword, promoting tumor progression and inducing drug resistance.

**Figure 2:**
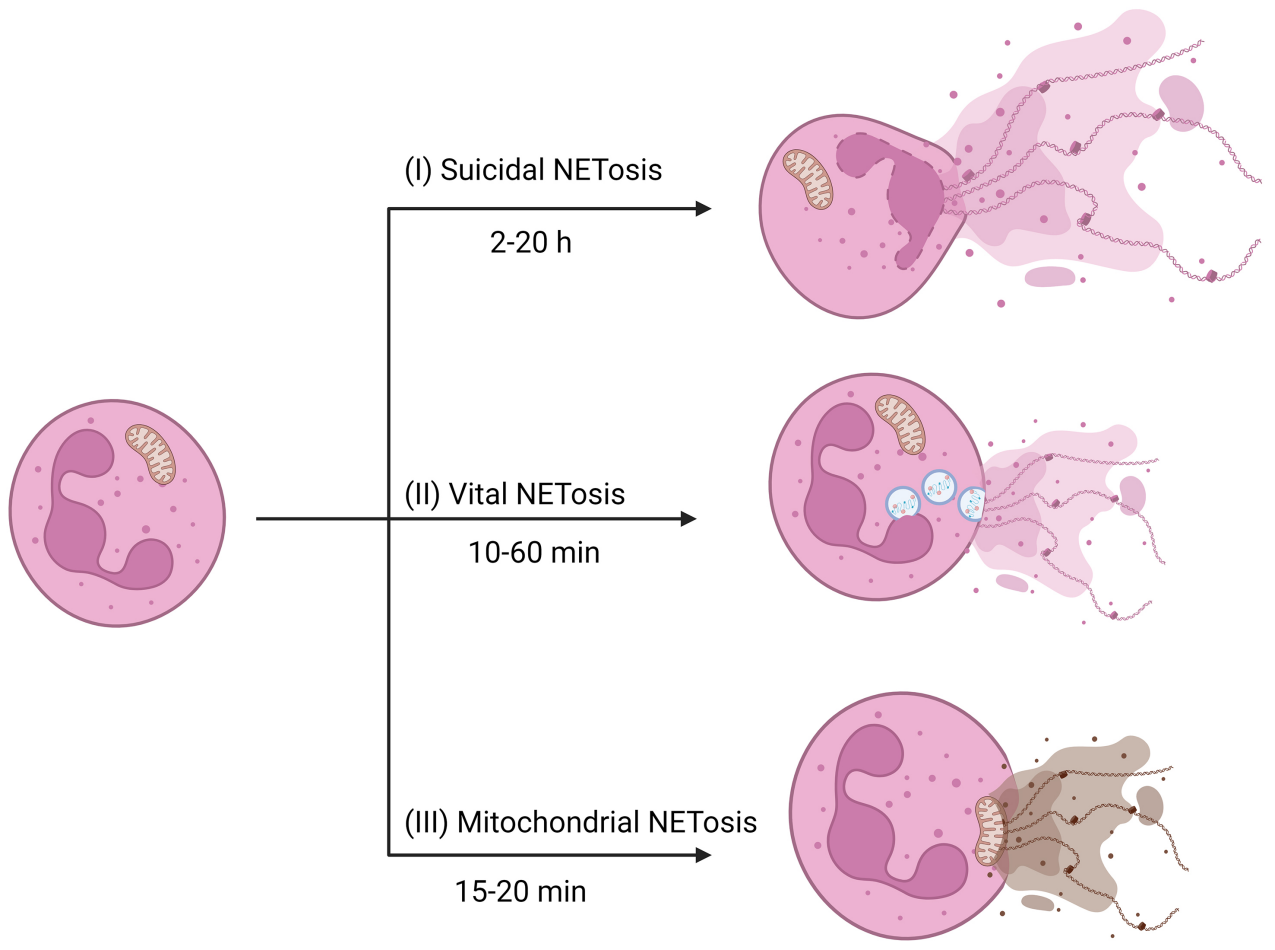
The schematic diagrams of three types of NETosis: Suicidal NETosis, Vital NETosis, and Mitochondrial NETosis. (I) Suicidal NETosis: Activated neutrophils undergo membrane rupture, releasing nuclear DNA and proteins into the extracellular space. The entire process takes several hours to complete, and following completion, the neutrophil undergoes cell death; (II) Vital NETosis: Activated neutrophils release nuclear DNA and proteins into the extracellular space through vesicular transport, completing the entire process within 10-60 min. Neutrophils remain viable after completing this process; (III) Mitochondrial NETosis: Activated neutrophils release mitochondrial DNA and proteins into the extracellular space, completing the entire process in only 15-20 min, with the neutrophils maintaining cellular viability after the process. Created with BioRender.com (accessed on 30 June 2022). NET: Neutrophils extracellular trap; DNA: deoxyribonucleic acid.

NETosis is a distinct mode of cell death in neutrophils, differing from apoptosis and necrosis, characterized by chromatin decondensation^[37]^. Three types of NETosis have been identified (suicidal, vital, and mitochondrial)^[38]^. Suicidal NETosis involves the release of NETs through nuclear membrane rupture, while vital NETosis transports nuclear DNA extracellularly via vesicle formation, and mitochondrial NETosis releases mitochondrial DNA to form NETs^[39]^. Neutrophils remain viable throughout the entire processes of the latter two types^[40]^. Furthermore, the time required for the formation of NETs varies among these three types: suicidal NETosis typically takes several hours after stimulation to produce NETs, vital NETosis can generate NETs within 10-60 min, and mitochondrial NETosis occurs even faster, forming NETs within 15-20 min^[41]^ [Figure 2 and Table 1].

**Table 1:** Comparison of three NETosis characteristics

	**Suicidal NETosis**	**Vital NETosis**	**Mitochondrial NETosis**
**Duration**	2-20 h	10-60 min	15-20 min
**Stimuli**	IL-8, PMA, bacterial endotoxins	Complements, microbes, active platelets	GM-CSF, LPS, C5a
**Receptors**	CXCR1/2, TLRs	Complement receptors, TLRs	To be identified GM-CSF receptor, TLR4, C5aR1
**ROS**	Dependent	Independent	Dependent
**DNA composition**	Nuclear	Nuclear	Mitochondrial
**PAD4**	Independent	Dependent	To be identified
**NE**	Dependent	To be identified	To be identified
**MPO**	Dependent	To be identified	To be identified
**Nuclear membrane**	Broken	Unbroken	Unbroken
**Cell membrane**	Broken	Unbroken	Unbroken
**Survival**	Death	Survival	Survival

IL-8: Interleukin-8; PMA: phorbol 12-myristate 13-acetate; GM-CSF: granulocyte/macrophage colony-stimulating factor; LPS: lipopolysaccharide; C5a: complement factor 5a; CXCR1/2: C-X-C chemokine receptor 1 and 2; TLRs: Toll-like receptors; TLR4: Toll-like receptor 4; C5aR1: complement factor 5a receptor 1; ROS: reactive oxygen species; DNA: deoxyribonucleic acid; PAD4: peptidylarginine deiminase 4; NE: neutrophil elastase; MPO: myeloperoxidase.

#### Suicidal NETosis

Suicidal NETosis is primarily characterized by the activation of NOX and the generation of reactive oxygen species (ROS)^[37]^. Interleukins, PMA, and bacterial endotoxins have been found to stimulate neutrophil activation. A study utilized 20 nM PMA to induce neutrophil activation to investigate the morphological changes associated with NETosis. It was observed that after 60 min, the neutrophil nuclei began to lose their lobulated structure, and chromatin started decondensing, while the nuclear envelope remained intact and the space between the inner and outer nuclear membranes expanded. After 120 min, the nuclear envelope formed distinct vesicles. By 180 min, the nuclear envelope had disintegrated into numerous small vesicles, chromatin was fully decondensed, and most granules had disappeared^[37]^. Upon binding of specific surface receptors on neutrophils with stimulants, the endoplasmic reticulum releases calcium ions, leading to the activation of protein kinase C (PKC) in response to increasing Ca^2+^ concentrations. Subsequently, the Raf-mitogen-activated protein kinase kinase (MEK)-ERK signaling pathway is activated^[42]^. With the activation of the NOX, ROS are generated. Additionally, IL-8 activates NF-κB via the C-X-C chemokine receptor 2 (CXCR2)-phosphatidylinositol 3-kinase (PI3K)-protein kinase B (AKT) signaling pathway, inducing inducible nitric oxide synthase (iNOS) and cyclooxygenase-2 (COX2) to increase ROS generation.

**Figure 3:**
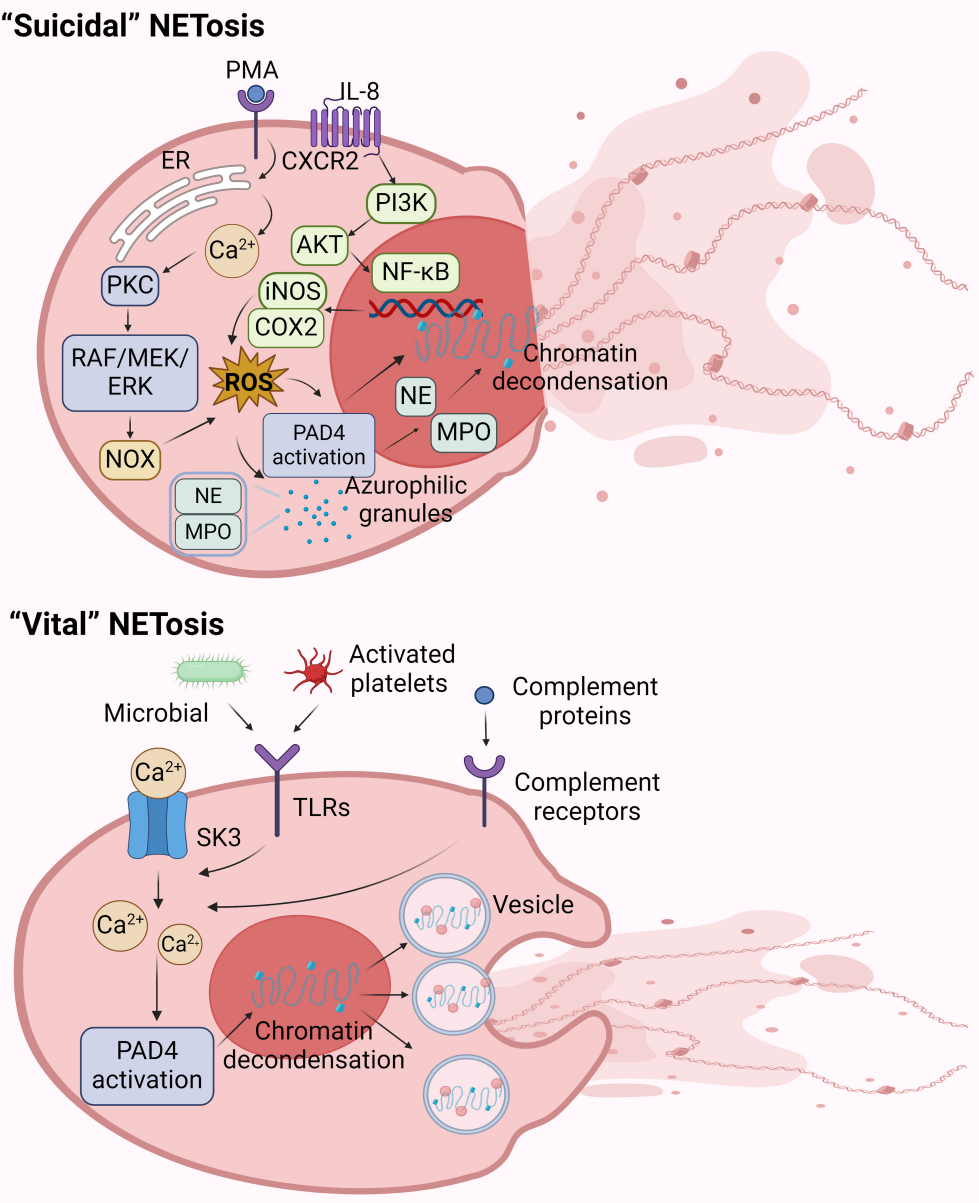
The mechanisms of “suicide” and “vital” NETosis. “Suicidal” NETosis can be induced by PMA or IL-8. PMA causes the release of Ca^2+^ from the ER and activation of PKC by binding to neutrophils’ surface receptors. Subsequently, the Raf-MEK-ERK signaling pathway is activated, leading to the activation of NOX and the elevation of intracellular ROS. Alternatively, IL-8 activates NF-κB by inducing the CXCR2-PI3K-AKT signaling pathway. Subsequently, iNOS and COX2 were induced to increase intracellular ROS. The increase in ROS allows NE and MPO to enter the nucleus from azurophilic granules. PAD4, on the other hand, is activated into the nucleus. NE, MPO, and PAD4 together promote chromatin decondensation. Finally, NETs are formed and released into the cell through cell membrane lysis. “Vital” NETosis can be induced by complement, microbial, and active platelet stimulation. In activated neutrophils, Ca^2+^ is transferred into the cell via SK3 channels and PAD4 is activated. Eventually, this results in the decompression of chromatin to form NETs that are transported out of the neutrophils via vesicles. Created with BioRender.com (accessed on 30 June 2022). PMA: Phorbol 12-myristate 13-acetate; IL-8: interleukin-8; ER: endoplasmic reticulum; PKC: protein kinase C; MEK: mitogen-activated protein kinase kinase; ERK: extracellular signal-regulated kinase; NOX: NADPH oxidase complex; ROS: reactive oxygen species; NF-κB: nuclear factor-kappa B; CXCR2: C-X-C chemokine receptor 2; PI3K: phosphatidylinositol 3-kinase; AKT: protein kinase B; iNOS: inducible nitric oxide synthase; COX2: cyclooxygenase-2; NE: neutrophil elastase; MPO: myeloperoxidase; PAD4: peptidylarginine deiminase 4; NETs: neutrophils extracellular traps; SK3: small conductance Ca^2+^ activated K^+^ channels.

The elevated ROS levels can activate several key proteins in NETosis. In resting neutrophils, NE and MPO are stored in azurophilic granules. Increased ROS levels stimulate the release of NE and MPO from azurophilic granules into the cell nucleus. NE, upon entering the cell nucleus, cleaves histones and induces chromatin decondensation^[43]^. On the other hand, elevated ROS levels activate peptidylarginine deiminase 4 (PAD4) enzyme, which, in the presence of Ca^2+^, mediates the conversion of arginine to citrulline on histones, further inducing chromatin decondensation^[44]^. Ultimately, this process leads to cell membrane rupture, the release of NETs into the extracellular space, and the demise of the neutrophil, marking the conclusion of the entire process. The rupture of the nuclear membrane and cell membrane is a morphological characteristic that distinguishes suicidal NETosis from the other two forms of NETosis [Figure 3].

#### Vital NETosis

Compared to suicidal NETosis, vital NETosis is a NOX-independent process unrelated to ROS generation. Activation of vital NETosis occurs through the interaction of complements, microbes, and active platelets with complement receptors or Toll-like receptors (TLRs) on the surface of neutrophils. After activation, Ca^2+^ can enter the cytoplasm through small conductance Ca^2+^ activated K^+^ channels (SK3 channels)^[45]^. The elevation of intracellular Ca^2+^ levels activate PAD4, promoting histone citrullination, resulting in a weakening of the electrostatic binding between histones and DNA, ultimately leading to chromatin decondensation and NET formation. NETs are transported from neutrophils to the extracellular space via vesicles, and neutrophils can continue their cellular functions without undergoing cell death during this process [Figure 3].

#### Mitochondrial NETosis

Mitochondrial NETosis, initially reported in 2009, is the process by which neutrophils release mitochondrial DNA (mtDNA) to form NETs^[40]^. Researchers stimulated neutrophils with granulocyte/macrophage colony-stimulating factor (GM-CSF) for 20 min, followed by a subsequent 15-minute stimulation with LPS or complement factor 5a (C5a), resulting in the release of NETs by neutrophils^[40]^. Notably, NETs formed in this manner contain mtDNA but lack nuclear DNA. The formation of these NETs depends on the production of ROS but does not lead to neutrophil death. Unfortunately, research on mitochondrial NETosis remains limited to date, and the conditions and mechanisms underlying its occurrence remain poorly understood.

## NETS AND TUMOR PROGRESSION: A LINEAR RELATIONSHIP

As research advances, there is a growing recognition of the significance of NETs in the TME. The potential biological roles of NETs in cancer, such as their involvement in antitumor activity, promoting tumor growth, metastasis, and therapeutic resistance, have initiated some exploratory investigations.

### NETs exert antitumor activity through cytotoxic effect

Currently, the majority of research focuses on the role of NETs in promoting tumor progression, with few studies considering the cytotoxic effect of NETs on tumor cells^[46-48]^. Recent evidence has documented the cytotoxic effect of NETs on human melanoma cells^[49]^. Researchers found that neutrophils release NETs in human ulcerative melanoma, but the quantity of intratumoral NETs is not associated with tumor progression. *In vitro* experiments demonstrated that co-culturing NETs with melanoma cells leads to cell necrosis. Additionally, NETs can bind to integrins on melanoma cells, thereby inhibiting tumor cell migration^[49]^. Another study demonstrated that melatonin can promote pancreatic cancer cells to secrete CXCL2, thereby recruiting neutrophils and inducing their transformation to the N1 phenotype. This process exerts an antitumor effect by inducing tumor cell apoptosis through NET-derived ROS^[50]^. Further research is needed to better elucidate the role and significance of NETosis in antitumor activity.

### NETs act as a driver of carcinogenesis and can promote tumor growth

**Figure 4:**
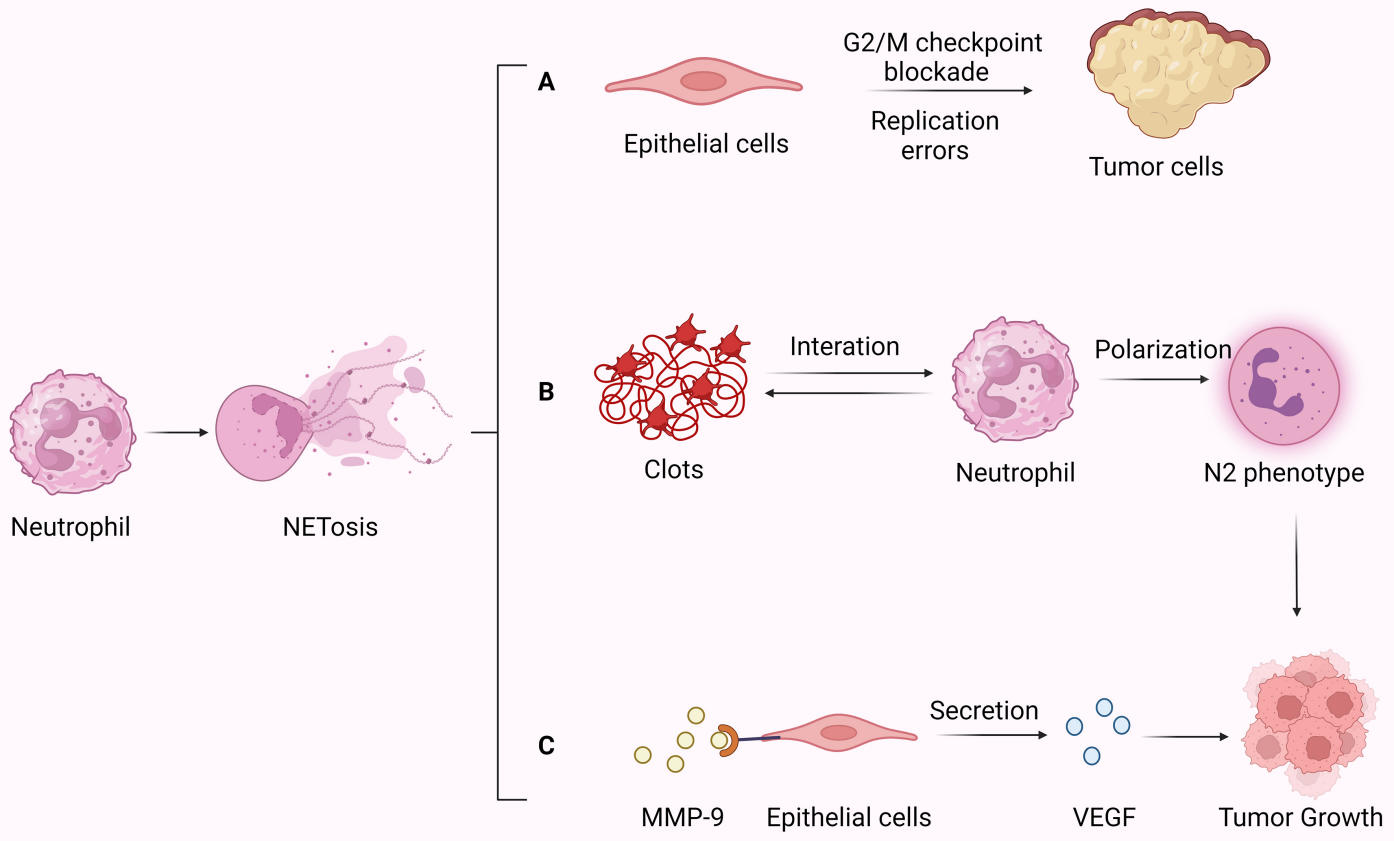
NETs act as a driver of carcinogenesis and can promote tumor growth. (A) NETs induce checkpoint blockade and replication errors in colon epithelial cells, leading to the onset of colorectal cancer; (B) The formation of clots by NETs with platelets reduces neutrophils’ function and induces their transformation to N2 phenotype; (C) MMP-9 acts on endothelial cells, promoting the secretion of VEGF, thereby further enhancing tumor growth. Created with BioRender.com (accessed on 30 June 2022). NETs: Neutrophils extracellular traps; MMP-9: matrix metalloproteinases-9; VEGF: vascular endothelial growth factors.

Neutrophils, when appropriately activated, play a crucial role in clearing invading pathogens. However, their sustained activation can lead to chronic pathological inflammation, such as autoimmune diseases, post-viral pneumonia^[51]^, and type 2 diabetes^[52]^. Persistent tissue damage and DNA injury resulting from NETosis are closely associated with cancer development. Studies have indicated that activated neutrophils observed in ulcerative colitis induce G2/M checkpoint blockade and replication errors in colon epithelial cells, ultimately leading to the onset of colorectal cancer^[53]^ [Figure 4A]. An increasing number of studies have demonstrated that neutrophils play a crucial role in arterial, venous, and cancer-associated thrombosis^[54]^, and are implicated in the occurrence and progression of pancreatic cancer^[55]^. Additional research has uncovered that in intestinal tumor-bearing mice, NETs provide a scaffold for the coagulation cascade, leading to the formation of clots that interact with circulating neutrophils. This interaction reduces neutrophils’ function and induces their transformation into the N2 phenotype, thereby promoting tumor growth^[56]^ [Figure 4B].

NETs can directly promote tumor growth by inducing cell proliferation and angiogenesis. They contain MMP-9, which triggers the release of vascular endothelial growth factor (VEGF) in pancreatic and lung cancer patients by degrading the extracellular matrix, thereby promoting tumor growth^[57]^ [Figure 4C]. In gastric cancer, its hypoxic microenvironment recruits and activates neutrophils via the TLR4/p38 MAPK signaling pathway, mediating NET formation. However, NETs can also induce tumor cell invasion and migration directly without inducing proliferation, thereby promoting cell growth by increasing angiogenesis^[58]^.

### NETs promote tumor metastasis

**Figure 5:**
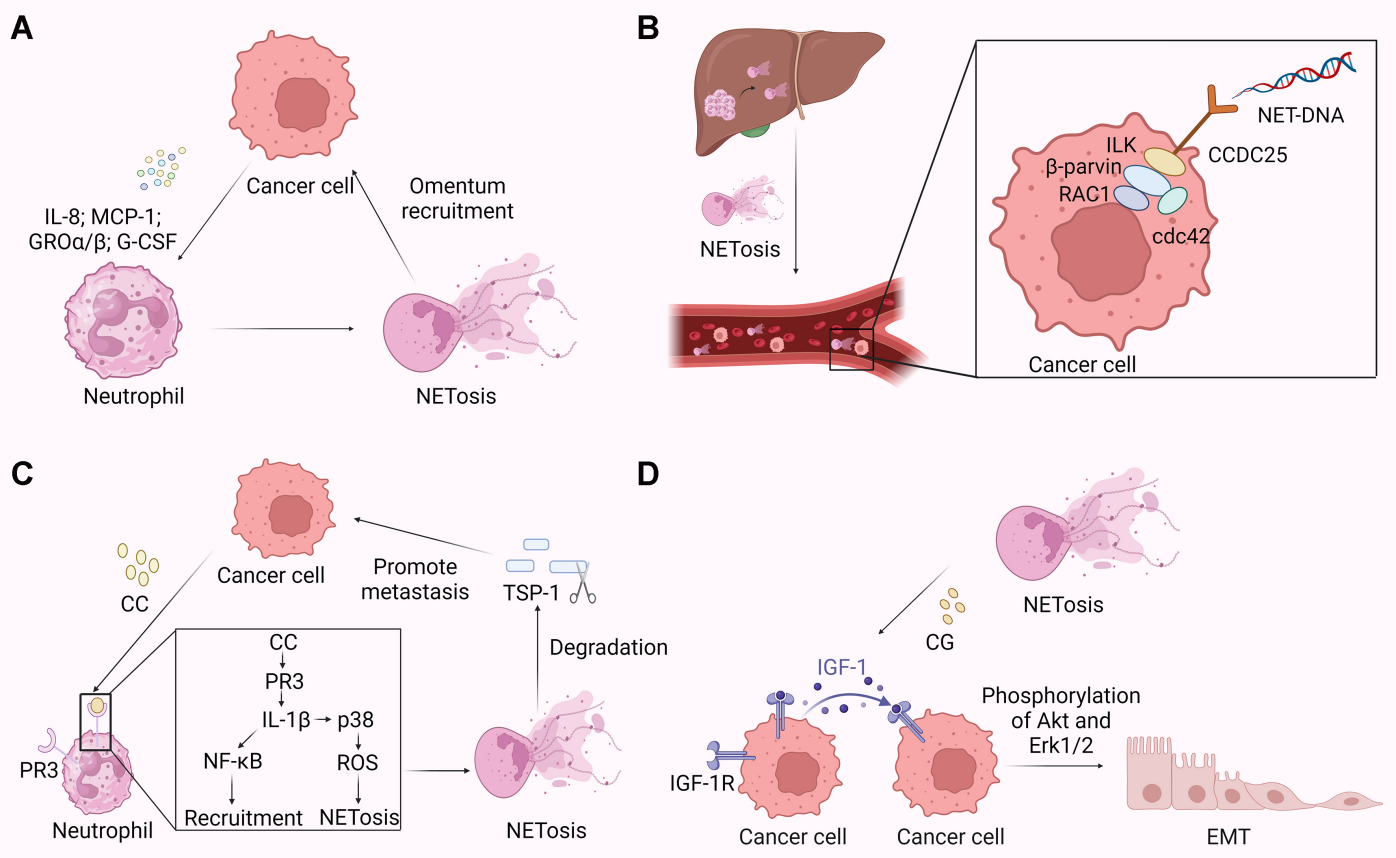
NETs promote tumor metastasis. (A) The mechanism of epithelial ovarian cancer intraperitoneal implantation. Neutrophils, attracted to the omentum by various chemotactic factors secreted by cancer cells, engage in NETosis, releasing chromatin that ensnares ovarian cancer cells, contributing to the formation of metastatic foci on the omentum; (B) The mechanism behind liver metastasis in breast and colon cancer. NET-DNA interacts with cancer cells by binding to CCDC25, initiating the ILK-β-parvin-RAC1-CDC42 signaling cascade within cancer cells; (C) The mechanism of lung metastasis in breast cancer. Tumor cells release CC, which interacts with PR3 on the neutrophil membrane, leading to neutrophil recruitment and triggering NETosis. Ultimately, this process promotes lung metastasis by degrading TSP-1; (D) The mechanism of EMT induced by NETs in tumor cells. CG stimulation releases IGF-1 from tumor cells, activating IGF-1R and inducing phosphorylation in MCF-7 cells. This activation enhances E-cadherin activity, subsequently stimulating secondary angiogenesis within target organs. Created with BioRender.com (accessed on 30 June 2022). NETs: Neutrophils extracellular traps; DNA: deoxyribonucleic acid; CCDC25: coiled-coil domain containing protein 25; ILK: integrin-linked kinase; RAC1: ras-related C3 botulinum toxin substrate 1; CDC42: cell division control protein 42; CC: cathepsin C; PR3: proteinase 3; TSP-1: thrombospondin-1; EMT: epithelial-mesenchymal transition; CG: cathepsin G; IGF-1: insulin-like growth factor-1; IGF-1R: insulin-like growth factor-1 receptor; MCF-7: Michigan Cancer Foundation-7.

NETs can promote tumor metastasis. A recognized feature of epithelial ovarian cancer is intraperitoneal implantation, with 60% of female patients having omental implants^[59,60]^, where neutrophils play a significant role. Neutrophils are recruited to the omentum by several neutrophil chemotactic factors secreted by cancer cells, including IL-8, monocyte chemoattractant protein-1 (MCP-1), growth-regulated oncogenes α and β (GROα and GROβ), and G-CSF. They are mobilized to undergo NETosis, releasing chromatin that captures ovarian cancer cells from the peritoneal fluid, thus forming metastatic foci on the omentum^[61]^ [Figure 5A]. NETs can capture liver cancer cells, and when internalized, they induce tumor cell invasiveness and trigger their metastatic potential by activating the TLR4/9-COX2 signaling pathway^[62]^. However, the mechanism by which NETs capture tumor cells remains unknown. Further exploration is needed to determine whether it occurs through the mesh structure of NETs trapping tumor cells or through specific ligand-receptor interactions between NETs and tumor cells.

Subsequently, a study elucidated the mechanism behind liver metastasis in breast and colon cancer. Prior to liver metastasis, patients’ livers produce a significant amount of NETs. NET-DNA can bind to coiled-coil domain containing protein 25 (CCDC25) on cancer cells, activating the integrin-linked kinase (ILK)-β-parvin-ras-related C3 botulinum toxin substrate 1 (RAC1)-cell division control protein 42 (CDC42) cascade within cancer cells^[63]^ [Figure 5B]. This cascade leads to actin remodeling and directional chemotaxis, thereby inducing cancer cell distant metastasis^[63]^. The study also proposed that the elevated levels of NETs in the blood could serve as a biomarker for early-stage cancer liver metastasis^[63]^.

Another study unveiled the role of neutrophils in promoting lung metastasis in breast cancer. Tumor cells secrete cathepsin C (CC) before metastasis, promoting neutrophil recruitment through the activation of the PR3-IL-1β-NF-κB axis, and inducing NETosis by activating p38 to generate reactive oxygen species. NETs, by degrading the metastasis-suppressive ECM protein [thrombospondin-1 protein (TSP-1)], facilitate lung metastasis of tumor cells^[64]^ [Figure 5C]. This study also assessed the clinical relevance of NETosis to breast cancer lung metastasis, revealing increased neutrophil infiltration and NETosis in lung metastases compared to primary tumors^[64]^.

NETs contain 583 distinct proteins^[65]^. In addition to the above-mentioned classic proteins, carcinoembryonic antigen-related cell adhesion molecule 1 (CEACAM1) was discovered to be structurally present on NETs and on the surface of activated neutrophils^[65-67]^. CEACAM1 on NETs plays a crucial role in the adhesion and migration of colon carcinoma cells. Furthermore, CEACAM1 on NETs is important for the adhesion of murine colon carcinoma cells to liver sinusoids *in vivo*. Blocking CEACAM1, using the monoclonal antibody 5F4, can significantly reduce the migration of colonic carcinoma cells^[65]^. These findings suggest that CEACAM1 plays a pivotal role in cancer cell metastasis.

Epithelial-mesenchymal transition (EMT) is a fundamental process in cancer where cancer cells detach from their surrounding tissues and seed new tumor lesions through the bloodstream^[68,69]^. Increasing evidence suggests that NETs can induce EMT in tumor cells, leading to tumor metastasis. Stimulation with CG triggers the release of insulin-like growth factor-1 (IGF-1) from tumor cells, which, via paracrine/autocrine signaling, activates the IGF-1 receptor (IGF-1R). This induction results in the phosphorylation of Akt and Erk1/2 in Michigan Cancer Foundation-7 (MCF-7) cells, further promoting the activation of E-cadherin^[70]^. E-cadherin-dependent cell-cell adhesion initiates cell migration, followed by the formation of cell aggregates, resulting in vascular and lymph node occlusion, stimulating secondary angiogenesis within target organs [Figure 5D]. This cascade promotes tumor metastasis and facilitates the growth of metastatic tumors^[71,72]^. Similarly, NE induces EMT through Src/PI3K-dependent Akt signaling, driving tumor cell migration^[73]^.

## THE ROLE OF NETs IN TUMOR TREATMENT RESISTANCE

### The role of NETs in chemotherapy resistance

**Figure 6:**
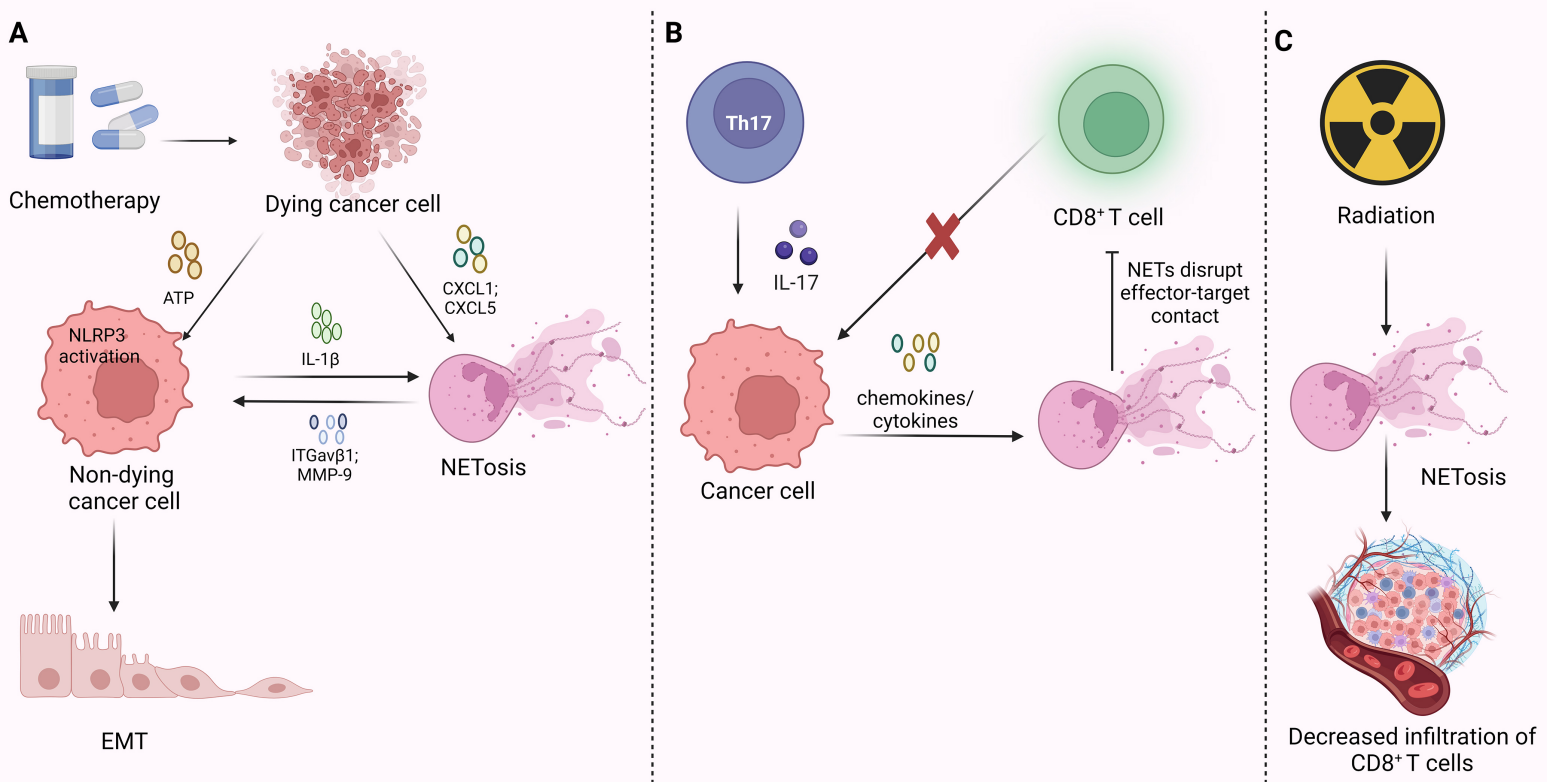
The role of NETs in tumor chemotherapy, immunotherapy, and radiation resistance. (A) Chemotherapy-induced tumor cell death induces NETosis in two ways: by secreting chemokines that directly induce NETosis or by releasing ATP, which prompts surviving tumor cells to secrete cytokines that further trigger NETosis. The NETs contain ITGαvβ1 and MMP-9, which can induce EMT and confer chemoresistance to tumor cells; (B) Th17 cells secrete IL-17, which induces tumor cells to release chemokines and cytokines, subsequently promoting NETosis. The components of NETs can inhibit immune-mediated cytotoxicity by disrupting the contact between effectors and their targets, leading to resistance to immunotherapy; (C) Radiation can induce NETosis, and inhibiting NETs can enhance CD8^+^ T cell infiltration. Created with BioRender.com (accessed on 30 June 2022). NETs: Neutrophils extracellular traps; ATP: adenosine triphosphate; ITGαvβ1: integrins αvβ1; MMP-9: matrix metalloproteinases-9; EMT: epithelial-mesenchymal transition; Th17: T helper 17; IL-17: interleukin-17.

Although neutrophils make up the majority of leukocytes in human blood and have established roles in the progression of cancer, their involvement in chemoresistance remains poorly understood. In a mouse model of multiple myeloma, researchers have provided the first evidence that the elimination of CD11b^+^ cells in the bone marrow significantly retards tumor growth and enhances responsiveness to chemotherapy. Conversely, the expansion of CD11b^+^ cells results in reduced mouse survival and chemotherapy sensitivity^[74]^. It has been revealed that PMN-MDSCs and neutrophils mediate a chemical protective effect on myeloma cells by secreting soluble factors, shielding them from the effects of amphotericin^[74]^. Research in 2019 underscored the non-classical functions of MPO enzyme in maintaining redox balance and mitochondrial energy metabolism^[75]^. Cytarabine (AraC) is a crucial chemotherapeutic regimen for treating acute myeloid leukemia (AML). However, AML cells with high MPO expression exhibit reduced sensitivity to AraC and produce fewer ROS^[75]^. MMP-9, released within NETs, serves as a critical driver of angiogenesis and metastasis in cancer. Chemotherapy-induced cell death in tumors leads to the secretion of CXCL1 and CXCL5, attracting neutrophils to the tumor site^[76]^. Simultaneously, dying tumor cells release adenosine triphosphate (ATP), inducing NLRP3 activation in surviving tumor cells, subsequently resulting in the release of IL-1β^[76]^. IL-1β can trigger neutrophils to secrete NETs. This study suggests that two proteins found in NETs, adenosine triphosphate; ITGαvβ1: integrins αvβ1 (ITGαvβ1) and MMP-9, play important roles^[76]^. The former captures TGF-β, while the latter cleaves and activates TGF-β, inducing TGF-β-dependent EMT and cisplatin or adriamycin/cyclophosphamide (AC) chemoresistance in tumor cells^[76]^ [Figure 6A]. CG induces the formation of cell clusters in MCF-7 breast cancer cells by triggering the insulin-like growth factor-1 signaling pathway, decreasing doxorubicin sensitivity^[77]^.

### The role of NETs in immunotherapy resistance

In recent years, immunotherapy has demonstrated significant efficacy in a variety of malignancies. Exemplifying this success are immune checkpoint inhibitors (ICIs), particularly programmed cell death protein 1 (PD-1) antibodies and their receptor, PD-L1 antibodies, which have shown promise in the treatment of various cancers. Increasing evidence now suggests that NETosis and the components of NETs play a role in immune therapy resistance.

A study has found that NETs contain the immunosuppressive ligand PD-L1, which can inhibit T cell responses through metabolic and functional exhaustion, thereby promoting tumor growth^[78]^. Some researchers have proposed the prognostic value of serum CEACAM1 in monitoring tumor burden and disease progression. They found that serum CEACAM1 levels are elevated in patients who do not respond to immunotherapy compared to those who exhibit responses and disease stabilization^[79]^. CEACAM1 forms a heterodimer with T-cell immunoglobulin domain and mucin domain-3 (TIM-3), an activation-induced immunosuppressive molecule. This interaction promotes TIM-3 maturation and cell surface expression, ultimately inducing T-cell exhaustion^[80]^.

Interleukin-17 (IL-17) is produced by T helper 17 (Th17) cells, CD8 T cells, γδT cells, and natural killer (NK) cells in the TME^[81]^. Research has revealed that IL-17 recruits neutrophils and induces NETosis in lung tumor-bearing mice, with a notable trend toward reduced T-cell counts correlating with increased disease burden, rendering tumors resistant to ICIs^[82]^. This phenomenon has been validated in a murine model of pancreatic ductal adenocarcinoma, where Th17 cells secreting IL-17 increase in tumor tissue. Through the IL17/IL17R signaling pathway, they induce the secretion of chemokines/cytokines by tumor cells, triggering the release of NETs while excluding cytotoxic CD8^+^ T cells from tumors^[83]^. Concurrently, another study confirmed the mechanism by which NETosis leads to immune resistance. Tumor cells secrete ligands for CXCR1 and CXCR2, such as CXCL1, CXCL2, and CXCL8, which induce neutrophils to undergo NETosis and release NETs. NETs disrupt effector-target contact, inhibiting immune-mediated cytotoxicity and thereby shielding tumor cells from the cytotoxic effects of CD8^+^ T cells and NK cells. This results in diminished efficacy of ICIs^[84]^ [Figure 6B].

### The role of NETs in RT resistance

RT offers treatment opportunities for patients who do not meet the criteria for surgical intervention or are averse to undergoing radical organ resection due to significant reductions in their quality of life. However, radioresistance of tumor cells remains a significant hurdle to effective treatment^[85]^. Neutrophils have been shown to promote resistance to RT, and in mouse sarcoma models, neutrophil depletion increased the radiosensitivity of tumors^[86]^.

Emerging evidence suggests a crucial role for NETs in treatment resistance^[85]^. Radiation can induce the formation of NETs, and inhibiting NETs enhances the efficacy of RT and increases the infiltration of CD8^+^ T cells in tumor foci^[85]^ [Figure 6C]. Researchers have also demonstrated clinical relevance, with a significantly higher proportion of non-responders to RT showing NETs in the tumor immune microenvironment (TIME) compared to RT responders, which is associated with poorer survival rates^[85]^. Currently, there is limited research on the role and mechanisms of NETs in RT resistance, and further exploration is required.

## POTENTIAL CLINICAL APPLICATIONS OF NETS IN CANCER

### NETs as biomarkers for cancer diagnosis

An increasing body of research suggests that biomarkers of NETosis in the bloodstream can serve as biomarkers for various cancers and predict adverse outcomes^[87]^. Elevated levels of NETs are associated with disease staging. Circulating NET levels are higher in late-stage esophageal, gastric, and lung cancer patients compared to local cancer patients and healthy individuals^[88]^. This study also demonstrated that higher NET levels in cancer patients can serve as a biomarker for progressive disease, independent of NLR or absolute neutrophil count, which are standard clinical biomarkers for cancer prognosis. In endometrial cancer (EC) patients, NETosis characterized by citH3 positivity was observed in all grades of tissue. Meanwhile, elevated citH3 and cell-free DNA (cfDNA) levels and decreased cell-free mitochondrial DNA (cfmtDNA) levels in serum can distinguish EC patients from healthy individuals^[88]^.

In late-stage cancer patients, plasma citH3 levels are higher and correlate with short-term mortality^[87]^. Another study confirmed that elevated citH3 and cfDNA levels are associated with higher mortality rates in cancer patients^[89]^. Researchers have developed an ICI treatment response prediction model based on serum levels of citH3, IL-8, and CRP, referred to as the “risk score” = 3.4591 × citH3 + 2.5808 × IL-8 + 2.0045 × CRP - 11.3844. The cutoff point for the “risk score” was 0.528, and patients with a “risk score” lower than 0.528 were more likely to benefit from ICI treatment^[89]^.

Proteins derived from NETs can interact with circulating DNA, and, as a result, in some studies, levels of circulating NET-derived DNA complexes, such as MPO-DNA and NE-DNA, have been considered as biomarkers for cancer. In a study involving pancreatic cancer (PAAD) patients, pre-treatment levels of total DNA and MPO-DNA showed a positive correlation with clinical staging^[90]^. Serum MPO-DNA levels have been confirmed as predictive factors for early breast cancer liver metastasis^[63]^. Patients with colorectal liver metastases (CRLM) who exhibited preoperative MPO-DNA levels above the median had significantly shorter disease-free survival (DFS) and overall survival (OS) compared to those with MPO-DNA concentrations below the median^[91]^. Research has indicated that NE-DNA is an independent prognostic factor for OS in gastric cancer patients^[92]^.

In summary, from a clinical perspective, NETs and their associated components play a fundamental role as cancer biomarkers. However, it is observed from numerous studies that the clinical application of NETs is limited at a technical level. Currently, there is a lack of uniform diagnostic criteria and standardized quantitative thresholds in clinical practice for the detection of NETs as biomarkers.

### NETs as a target for cancer therapy

In light of the aforementioned studies, NETs have been closely associated with higher mortality rates and adverse outcomes in cancer patients. Pathways involved in inhibiting the formation of NETs could serve as potential therapeutic targets for controlling cancer progression and metastasis. Experimental and preclinical studies are underway to degrade pre-existing NETs and prevent abnormal NET formation. Target molecules can be categorized into four main groups: NET components, chemokines, transmembrane receptors, and cytokines [Table 2].

**Table 2:** List of studies targeting NETs in various cancer

**Target molecules**	**Mechanism**	**Agent**	**Disease**	**Ref.**
**NETs components**				
DNA	Degrading formed NETs	DNase I	Pancreatic cancer	[77]
			Colorectal cancer	[82]
			Hepatocellular carcinoma	[50]
			Breast cancer	[52,71]
PAD4	Inhibitor of PAD4	GSK484	Breast cancer	[52,71]
			Hepatocellular carcinoma	[84]
			Melanoma	[85]
			Ovarian cancer	[49]
		Cl-amidine	Melanoma	[85]
			Ovarian cancer	[49]
		BMS-P5	Multiple myeloma	[86]
		JBI-589	Lung cancer Colon cancer	[87]
NE	Inhibitor of NE	GW311616	Diffuse large B-cell lymphoma	[88]
Cathepsin C	Inhibitor of cathepsin C	AZD7986	Breast cancer	[92]
**Chemokine receptor**				
CXCR1/2	Inhibitor of CXCR1/2	Reparixin	HER-2 negative breast cancer	[94]
		SX-682	Head and neck squamous cell carcinoma	[95]
**Transmembrane receptor**				
PR3	Inhibitor of PR3	Sivelestat	Breast cancer	[52]
CCDC25	Anti CCDC25	Anti-CCDC25 antibody	Breast cancer	[51]
TLR9	Antagonist of TLR9	ODN-TTAGGG	Diffuse large B-cell lymphoma	[88]
**Cytokines**				
IL-1β	Anti IL-1β	Anti IL-1β antibody	Breast cancer	[64]
IL-17	Anti IL-17	Anti IL-17 antibody	Pancreatic ductal adenocarcinoma	[70]

NETs: Neutrophils extracellular traps; DNA: deoxyribonucleic acid; PAD4: peptidylarginine deiminase 4; NE: neutrophil elastase; CXCR1/2: C-X-C chemokine receptor 1 and 2; HER-2: human epidermal growth factor receptor 2; PR3: proteinase 3; CCDC25: coiled-coil domain containing protein 25; TLR9: Toll-like receptor-9; ODN: oligodeoxynucleotides; IL-1β: interleukin-1β; IL-17: interleukin-17.

#### Components of NETs as targets

DNase I is a nucleic acid endonuclease that cleaves the core component DNA of NETs^[93]^. Currently, the Food and Drug Administration (FDA) has approved DNase I for the treatment of cystic fibrosis^[94]^. Treatment of mice with DNase I (5 mg/kg IP daily) that carried PAAD resulted in a significant reduction in tumor growth rate compared to the control group treated with a sham procedure (pancreatic weight: 336 ± 26.4 mg *vs.* 206 ± 31.6 mg)^[90]^. An adeno-associated virus gene therapy vector for DNase I expression, AAV DNase I, when administered, reduced neutrophil recruitment and inhibited NET formation in human tumor cells, ultimately suppressing the growth of colorectal cancer liver metastases^[95]^. NETs promote the metastasis of liver cell carcinoma by triggering a tumor inflammatory response. The combined use of DNase I with the anti-inflammatory drugs aspirin and hydroxychloroquine effectively reduced intrahepatic and pulmonary metastases in an HCC mouse model^[62]^. DNase I treatment inhibited breast cancer spheroid growth^[64]^ and pulmonary metastasis in breast cancer^[84]^.

GSK484, a highly specific small molecule inhibitor of PAD4^[96]^, inhibits lung metastasis and NETosis induced by overexpressed CC in AT3 breast cancer cells^[64]^. Compared to the untreated group, the PAD4 inhibitor GSK484 treatment group shows a reduction in the number of lung micro-metastases in BALB/C mice 24 h after intravenous injection of mCherry^+^ 4T1 cells^[84]^. Consistent results are observed in the study of liver cancer lung metastasis^[97]^. Cl-amidine, a pan-PAD inhibitor, exhibits similar effects to GSK484 in inhibiting melanoma growth^[98]^. Inhibition of NET formation by treatment with GSK484 or Cl-amidine decreases omental colonization^[98]^. The novel inhibitor BMS-P5 can block the formation of neutrophil extracellular traps and delay the progression of multiple myeloma^[99]^. JBI-589, a novel PAD4 isoform-selective small molecule inhibitor, disrupts neutrophil chemotaxis, inhibits primary tumor growth and lung metastasis in mouse models, and enhances sensitivity to ICIs^[100]^. In summary, these results suggest the therapeutic potential of targeting PAD4 in cancer.

GW311616, an NE inhibitor, suppresses diffuse large B-cell lymphoma (DLBCL) cell proliferation and reduces axillary lymph node metastasis^[101]^. The MMP-9 inhibitor enalapril, when combined with 5-FU, enhances the sensitivity of colorectal cancer patients to 5-FU by cooperatively inhibiting the NF-κB/STAT3 signaling pathway^[102]^. AZD7986, an inhibitor of CC^[103]^, emerges as a candidate therapeutic agent for neutrophil-driven inflammatory diseases like chronic obstructive pulmonary disease (COPD)^[104,105]^. Oral administration of AZD7986 in immune-deficient mice bearing intravenous MDA231-LM2 cells (highly metastatic human breast cancer cells) significantly inhibits mouse circulation and lung NETs while alleviating lung metastasis^[105]^.

#### Chemokine receptors as targets

Activation of the surface receptors CXCR1/2 on neutrophils can induce NETosis. Numerous clinical trials are currently investigating the combination of CXCR1/2 inhibitors with ICIs as a strategy to mitigate NETosis. Among these, Reparixin, a CXCR1/2 allosteric inhibitor, has demonstrated promising activity against breast cancer stem cells when used in combination with paclitaxel in a phase Ib clinical trial for human epidermal growth factor receptor 2 (HER-2) negative metastatic patients (NCT02370238)^[106]^. SX-682, an orally bioavailable small-molecule allosteric inhibitor of CXCR1/2, is undergoing clinical evaluation (NCT03161431)^[107]^. Research indicates that the mechanism of action of this inhibitor may involve reducing tumor accumulation of CXCR2^+^ MDSCs in mice, enhancing the efficacy of T cell immunotherapy^[108]^, or increasing the tumor accumulation and activation status of KIL cells (a cultured murine NK cell line) for adoptive transfer by inhibiting PMN-MDSC trafficking^[107]^.

#### Transmembrane receptors as targets

Sivelestat, an inhibitor of PR3^[109]^, effectively mitigated CC-induced neutrophil recruitment and NETosis^[64]^. Blocking CCDC25, a receptor for NET-DNA, with polyclonal antibodies, efficiently inhibited NETs-induced cell migration *in vitro* and suppressed the hepatic metastasis of MDA-MB-231 cells in NOD/SCID mice *in vivo*^[63]^. Antagonists of TLR9 can eliminate the proliferation and migration of DLBCL cells driven by NETs^[101]^.

#### Cytokine as targets

The presence of IL-1β can induce NETosis, while the formation of NETs can trigger TGF-β-dependent EMT and resistance to chemotherapy in tumor cells. The use of IL-1β blocking antibodies inhibits the generation of NETs, reduces the aggregation of neutrophils, and decreases the number of metastatic foci in the lungs after chemotherapy^[76]^. Blocking IL17-triggered NETosis has shown significant antitumor effects in a murine pancreatic cancer model, enhancing the sensitivity to ICIs (PD-1, CTLA4)^[83]^.

### Targeting NETs with nano-platforms or liposomes for cancer treatment

Degradation of NETs with DNase I has been shown to enhance the sensitivity of tumor immunotherapy and inhibit tumor metastasis. However, DNase I has a short half-life in the bloodstream, and its systemic distribution may compromise the host’s defense against infections. Therefore, targeted delivery and controlled release of DNase I are of paramount importance. This concept can be achieved through the use of a nano-platform for DNase I loading.

**Figure 7:**
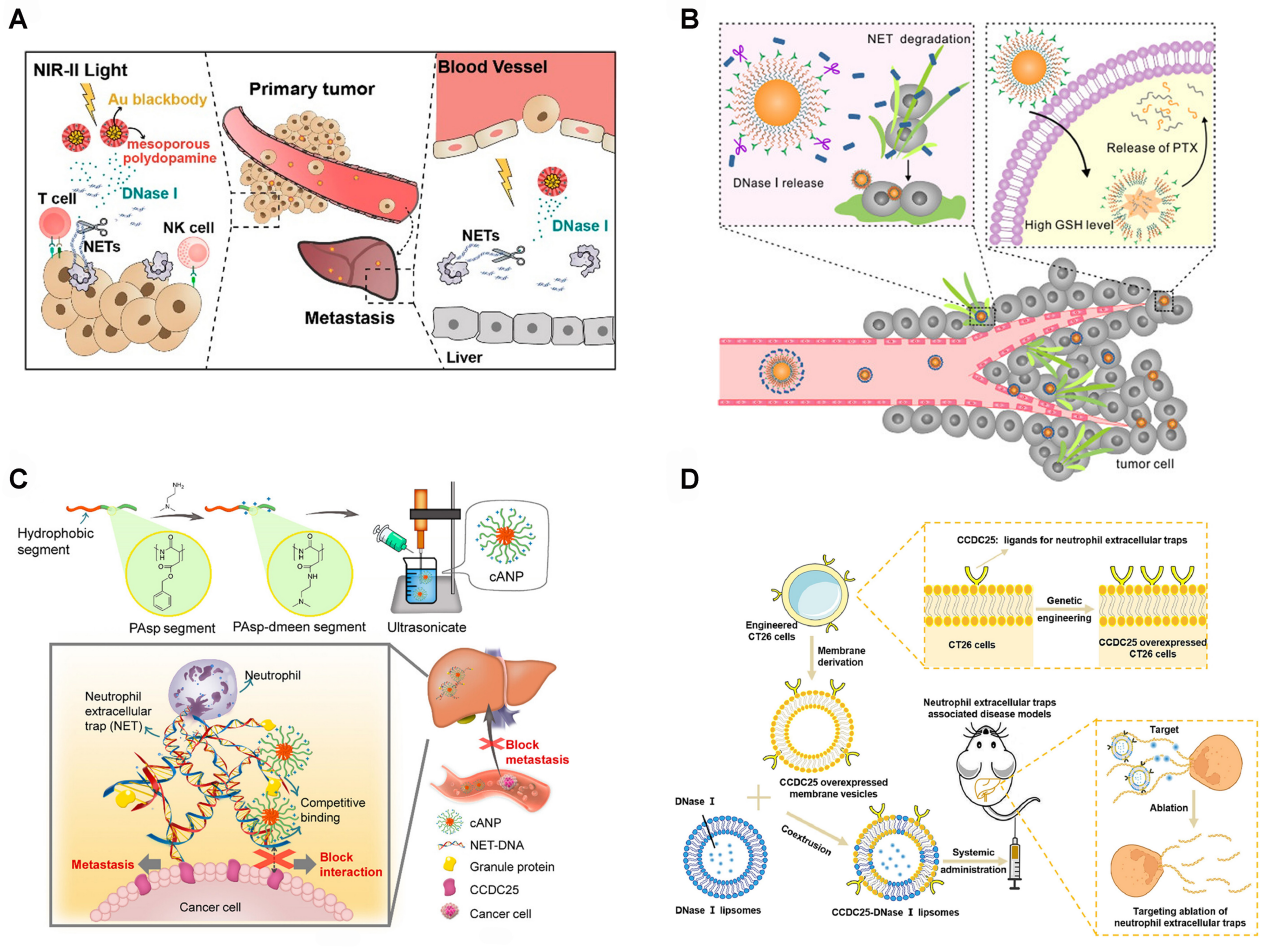
Schematic representation of targeted tumor therapy using nanoplatforms. (A) Schematic illustration of NIR-II-responsive AuPB@mPDA-DNase-mediated NETs degradation. By releasing DNase I to degrade NETs, this approach enhances the anticancer efficacy of immunotherapy and prevents liver metastasis; (B) GSH-responsive mP-NPs-DNase/PTX nanomaterials release PTX and DNase I, inhibiting tumor growth and distant metastasis; (C) Diagram showing cANP preparation and the use of cationic polymers to selectively bind NET-DNA, inhibiting cancer metastasis to the liver; (D) Diagram depicting cell membrane hybrid liposomes engineered through genetic modification for the specific elimination of NETs through CCDC25 targeting of NET-DNA and the action of DNase I. These figures are quoted with permission^[110-113]^. NIR-II: second near-infrared; NET: neutrophils extracellular trap; GSH: glutathione; PTX: paclitaxel; cANP: the poly (aspartic acid)-based cationic nanoparticle; DNA: deoxyribonucleic acid; CCDC25: coiled-coil domain containing protein 25.

Researchers have developed Au-PB@mPDA, a core-shell nanoparticle delivery system consisting of plasmonic gold-black (Au-PB) cores and mesoporous polydopamine (MPDA) shells. This system is designed for efficient loading and photo-regulated release of DNase I within the second near-infrared (NIR-II) spectral window. This nanoparticle enables the on-demand release of DNase I with precise spatiotemporal control, eliminating NETs in gastric cancer and the liver^[110]^. This results in increased sensitivity of gastric cancer cells to ICI therapy and reduced liver metastasis [Figure 7A]. Other researchers developed an intelligent nanoplatform loaded with DNase I, designated as mP-NPs-DNase/paclitaxel (PTX). The core of the nanocarrier is self-assembled from PTX prodrug nanoparticles. DNase I and a substrate peptide of MMP-9 were covalently linked to the polypeptide sequence of the cell-penetrating peptide (Tat) to serve as the shell. Upon reaching the tumor site, MMP-9 cleaved the substrate peptide, triggering the stimuli-responsive release of DNase I from mP-NPs-DNase/PTX. This efficient release led to the degradation of NETs. The remaining moiety of the nanocarrier could be internalized into tumor cells through Tat peptide. Subsequently, the high concentration of glutathione (GSH) within tumor cells caused the disulfide bonds in the nanoparticle core to break, releasing PTX and promoting the inhibition of tumor cell proliferation. mP-NPs-DNase/PTX serves as a potential nanocarrier for NET regulation, inhibiting tumor growth and distant metastasis [Figure 7B]^[111]^.

The poly (aspartic acid)-based cationic nanoparticle (cANP) exhibits strong electrostatic interactions with DNA and, by blocking the binding of NET-DNA to CCDC25, inhibits liver metastasis in a mouse breast cancer model^[112]^ [Figure 7C]. In another study, researchers engineered the CT26 cells to stably express CCDC25. Subsequently, they collected these cell membranes and fused them with liposomes loaded with DNase I, forming hybrid liposomes. CCDC25-DNase I liposomes efficiently degraded NETs and inhibited the aggregation of neutrophils, further suppressing liver metastasis in colorectal cancer^[113]^ [Figure 7D].

### BOX 1. Neutrophils as drug delivery carriers for tumor therapy through the release of NETs

**Figure 8:**
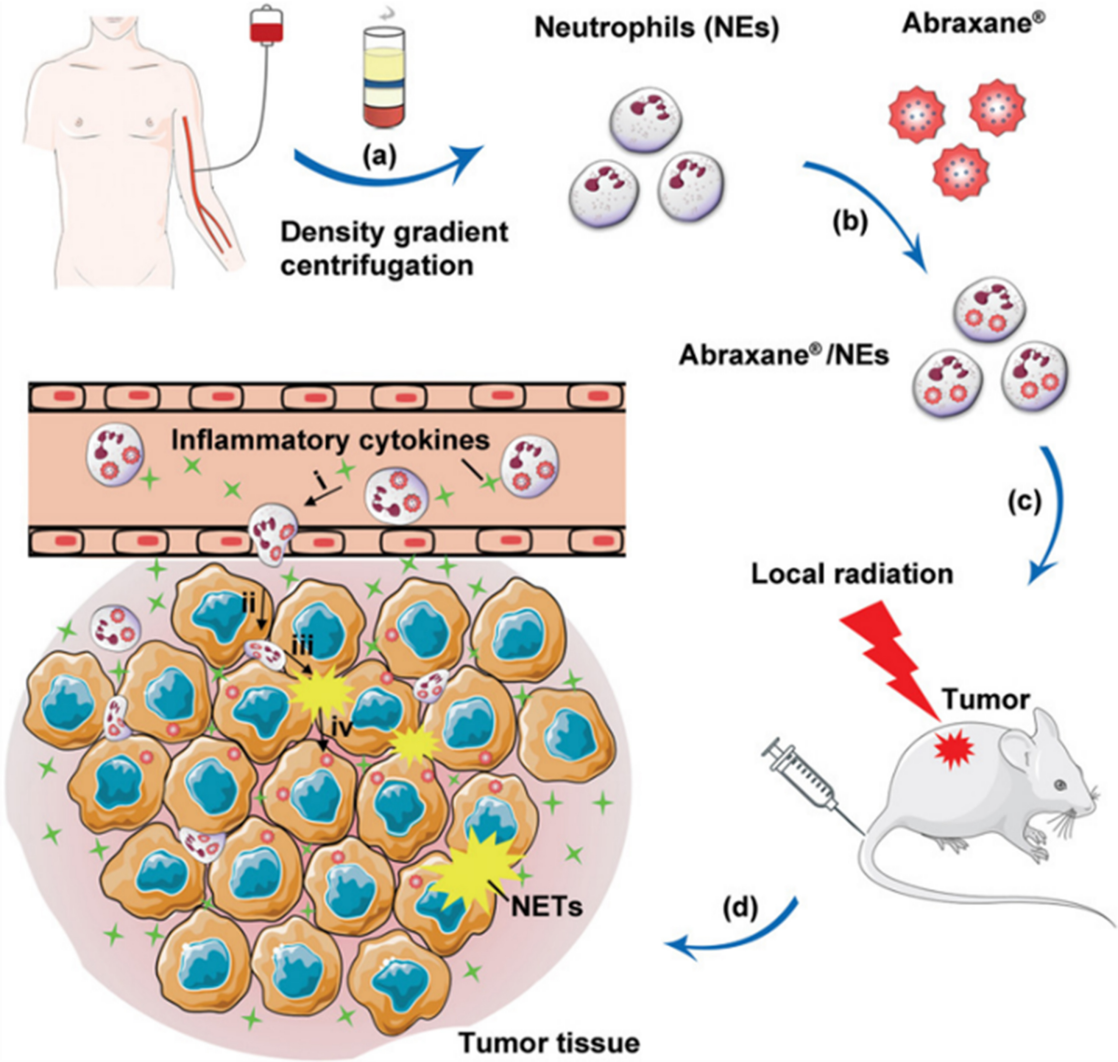
Illustration of how neutrophils act as drug delivery carriers, releasing therapeutic agents via NETs for the treatment of tumors. This figure is quoted with permission^[114]^. NETs: Neutrophils extracellular traps.

As tumors progress, neutrophils can be recruited into tumor tissues. Leveraging this phenomenon, researchers have employed neutrophils as drug delivery carriers, subsequently internalizing albumin-bound paclitaxel nanoparticles (Abraxane) to form cell-drug conjugates (Abraxane®/NEs). Combined with radiotherapy, neutrophil overactivation is induced, triggering NETosis, resulting in the formation of NETs and concurrent release of Abraxane. This nanoparticle can be internalized by tumor cells, inhibiting tumor cell mitosis and achieving an anticancer effect^[114]^ [Figure 8].

### Conclusion and future prospects

The duality of neutrophil phenotypes underscores the Janus-faced role of neutrophils in tumorigenesis and development. In the early stages of tumor development, N1 TANs play a prominent role, leveraging their innate defense mechanisms to exert antitumor effects. However, as the disease progresses, an increased infiltration of neutrophils occurs, leading to their polarization into the N2 phenotype in the TME, promoting tumor progression. With the escalating inflammation, NETosis is triggered, further enhancing tumor cell proliferation, migration, and invasion.

An increasing number of research indicates a significant association between the occurrence of NETosis or the composition of NETs and treatment resistance in tumor cells. The net-like structure of NETs and the activity of various related proteins or factors in the TME can reduce the efficacy of chemotherapy, immunotherapy, and radiotherapy. Nevertheless, the underlying mechanisms of NETs in radiotherapy resistance lack comprehensive clinical and foundational scientific data, which may serve as a direction for future research.

Given these, NETs hold potential clinical applications in the treatment of refractory cancers. NETs can serve as biomarkers for cancer development, showing a positive correlation with clinical staging and adverse prognosis. Presently, there is no consistent set of detection criteria and quantifiable thresholds in clinical settings to assess NETs as cancer treatment indicators. Pathways associated with inhibiting NET formation may offer potential therapeutic targets for cancer, and research has utilized nano-platforms for targeted drug delivery and controlled release. Regrettably, the optimal approach to combating NETs remains undetermined, and there are no FDA-approved drugs specifically designed for targeting NETs in cancer treatment. Basic research in the field should focus on regulating NETs and achieving a balance between NET formation and degradation without interfering with immune system functionality in the future. Clinical studies can further validate the efficacy of targeting NETs in inhibiting tumor growth.

Taken together, this review provides an overview of the role of neutrophils in the TME and their defense mechanisms. We also elucidate the mechanisms by which NETs promote tumor progression and their emerging role in cancer treatment resistance, emphasizing their potential as targets for cancer therapy and laying the theoretical groundwork for clinical trials utilizing these targets.

## DECLARATIONS

### Copyright

© The Author(s) 2024.
